# ASSESSING REAL-WORLD WALKING IN OLDER ADULTS LIVING WITH COPD

**DOI:** 10.1093/geroni/igae098.0742

**Published:** 2024-12-31

**Authors:** Laura Delgado-Ortiz, Clemens Becker, Thierry Troosters, Judith Garcia-Aymerich

**Affiliations:** Barcelona Institute for Global Health, Barcelona, Catalonia, Spain; Robert-Bosch-Hospital Stuttgart, Stuttgart, Baden-Wurttemberg, Germany; KU Leuven, Leuven, West-Vlaanderen, Belgium; IS Global, Barcelona, Catalonia, Spain

## Abstract

Walking is crucial for active and healthy ageing, but changes with age and in the presence of chronic conditions. In the field of chronic obstructive pulmonary disease (COPD) – a highly prevalent chronic condition at older ages - extensive research has been done on physical activity limitation, but little is known about the patterns of walking during daily life (i.e., real-world). We aimed to assess the levels and distribution of real-world walking in people with COPD compared with healthy adults, and across disease severity; and to identify domains of walking in COPD, using digital technology. We studied cross-sectionally 550 older adults with COPD and 19 healthy older adults from ten cities across eight countries. We derived twenty-five digital mobility outcomes (DMOs) that assess different characteristics of walking activity and gait (e.g., walking duration, number of walking bouts of different durations, walking speed, cadence, stride length variability) from a single device worn at subjects’ lower back for one week. Participants also answered validated questionnaires, and conducted lung function and exercise capacity tests. Most DMOs were reduced in older adults with COPD compared to healthy adults, and according to COPD severity. Differences remained statistically significant after adjusting for age, sex, height and comorbidities in multivariable analyses. Factor analysis identified six distinct walking domains: Amount of walking, Patterns of walking, Pace, Rhythm, Pace variability and Rhythm variability. Real-world walking in older adults with COPD can be characterised in multiple dimensions, is impaired in relation to their healthy peers, and worsens as disease progresses.

